# Role of serotonin in fatty acid-induced non-alcoholic fatty liver disease in mice

**DOI:** 10.1186/1471-230X-13-169

**Published:** 2013-12-09

**Authors:** Yvonne Ritze, Maureen Böhle, Synia Haub, Astrid Hubert, Paul Enck, Stephan Zipfel, Stephan C Bischoff

**Affiliations:** 1 Department of Nutritional Medicine, University of Hohenheim, Fruwirthstr. 12, 70599, Stuttgart, Germany; 2 Department of Psychosomatic Medicine, University of Tübingen, Tübingen 72076, Germany

**Keywords:** Non-alcoholic fatty liver disease, Saturated, Monounsaturated, Polyunsaturated fatty acids in diet, Serotonin, Intestinal permeability

## Abstract

**Background:**

Saturated fatty acids are thought to be of relevance for the development of non-alcoholic fatty liver disease and obesity. However, the underlying mechanisms are poorly understood. In previous studies we found that food-derived carbohydrates such as fructose alter the intestinal serotonergic system while inducing fatty liver disease in mice. Here, we examined the effect of fatty acid quantity (11% versus 15%) and quality (saturated, monounsaturated, or polyunsaturated fatty acids) on hepatic fat accumulation, intestinal barrier and the intestinal serotonergic system.

**Methods:**

C57BL/6 mice had free access to diets enriched with one of the three fatty acids or standard diet, for 8 weeks. In an additional experiment mice were fed diets enriched with saturated, monounsaturated fatty acids or standard diet supplemented with tryptophan (0.4 g/(kg^.^d), 8 weeks) or not. Hepatic fat accumulation, small intestinal barrier impairment and components of the serotonergic system were measured with RT-PCR, western blot or immunoassays. For statistical analysis t-test and one-way ANOVA with Tukey’s post hoc test and Bartlett’s test for equal variances was used.

**Results:**

Hepatic triglycerides, liver weight and liver to body weight ratio were significantly changed depending on the fat quality but not fat quantity. In contrast, fat quantity but not quality decreased the expression of the tight junction proteins occludin and claudin-1 in the small intestine. These changes seemed to result in enhanced portal vein endotoxin concentrations and fatty liver disease after feeding diet enriched with saturated and monounsaturated fatty acids but not polyunsaturated fatty acids. Neither fatty acid quantity nor quality significantly influenced the intestinal serotonergic system. Similarly, tryptophan supplementation had no impact on small intestinal barrier or fatty liver disease.

**Conclusion:**

In conclusion, diets rich in saturated or monounsaturated fatty acids promote the development of fatty liver disease in mice, likely by a dysfunction of the small intestinal mucosal barrier.

## Background

Non-alcoholic fatty liver disease (NAFLD) is one of the most important forms of liver disease in the developed countries, occurring in 20% to 25% of the general population [1,2]. Diet and lifestyle habits leading to obesity and insulin resistance are believed to exert the main pathophysiological role in the development of NAFLD [3-5]. Among the dietary factors, high fat intake and high sugar intake (“Western-style diet”) are thought to be of particular relevance [6]. In previous studies performed in mice, we could show that within the sugars, fructose plays a particular role for the development of NAFLD, whereas glucose is more relevant with regard to body weight [7]. Other studies suggested that a high fat intake, for example saturated fatty acids (SFA), is critical for both, the development of obesity and NAFLD [6,8,9]. In contrast, monounsaturated fatty acids (MUFA) and polyunsaturated fatty acids (PUFA) have been attributed rather to a healthy diet [10].

The mechanisms of diet-induced NAFLD are by far not clear at present. Based on previously published studies, we and others hypothesized that one possible mechanism of NAFLD development is an altered gastrointestinal (GI) barrier function. GI barrier dysfunction may be caused by particular diets or dietary components resulting in an impaired mucosal barrier and an elevated portal endotoxin level [11]. The endotoxin enters the liver where it is recognized by Toll-like receptors (TLR) and triggers innate immune responses and inflammation [12-14]. Evidence is increasing that high intake of dietary sugars may lead to increased portal endotoxin levels and as a result activation of TLRs [15]; however, the mechanisms by which high intake of dietary fatty acids cause obesity and fatty liver disease remain elusive.

Apart from the “translocation hypothesis”, stating that an impaired GI barrier leads to translocation of endotoxin from the gut lumen to the liver and causing fatty liver disease, other mechanisms might be considered. For example, serotonin (5-HT) derived from enterochromaffin cells, has been recognized as a potent pro-inflammatory mediator [16] and major regulator of intestinal permeability [14]. Interestingly, we could show that 5-HT is up-regulated in mice fed a fructose-rich diet and that liver steatosis induced by such a diet can be attenuated by suppressing 5-HT signaling [17]. The precursor of 5-HT is the amino acid tryptophan (TRP), which is also known to be a regulator of intestinal motility [18,19]. TRP has been used to alter the intestinal serotonin homeostasis, since intra peritoneal treatment with TRP enhances 5-HT levels [20,21]. Surprisingly, it also has been reported that TRP is capable of reducing hepatic lipid accumulation in particular animal models [22,23] and plasma triglycerides in humans [24]. In addition, TRP is able to exert anti-inflammatory effects in the liver [25].

Here, we studied potential mechanisms leading to the development of diet-induced fatty liver disease. We fed three types of fatty acid enriched diets with or without TRP supplement and investigated the effect of these diets on the intestinal barrier function, fatty liver disease and the intestinal serotonergic system in mice.

## Methods

### Mice and treatments

Mice were housed in a pathogen-free barrier facility accredited by the Association for Assessment and Accreditation for Laboratory Animal Care International (AAALAC). All procedures were approved by the local Institutional Animal Care and Use Committee (Regional Council Stuttgart). Female 6 weeks old C57BL/6 mice (Janvier, Le-Genes-St-isle, France) had ad libitum access, for 8 weeks, to either a modified (11% fat) standard diet (MZ-Ereich, Sniff®-Spezialdiäten GmbH, Soest, Germany) as control (C; 17.2 kJ/g diet) or 3 different diets enriched with fatty acids (15%) either SFA (beef tallow), MUFA (olive oil with a 14.4:1.6:1(n-9), (n-6), (n-7) fatty acid ratio), or PUFA (safflower oil with a 6:2 (n-6), (n-9) fatty acid ratio; *n* = 5 mice per group) (Table 1). The diets enriched with fatty acids were iso-energetic with 17.6 kJ/g diet (Table 2). In an additional experiment mice were fed a modified (11% fat) standard diet (MZ-Ereich, Sniff®-Spezialdiäten GmbH, Soest, Germany), the modified standard diet enriched with TRP (about 0.4 g/kg body weight); 0.24% w/w; C + TRP), a diet enriched with SFA or MUFA supplemented with TRP or not (*n* = 6 mice per group). The amount of TRP administered was selected according to Rogers et al. [23] (Table 1). Body weight, diet and fluid intake were assessed weekly. After 8 weeks, mice were anesthetized [7]. Blood was collected from the portal vein prior to killing. Small intestinal motility was assessed by the charcoal test. A charcoal solution (0.5 g charcoal; 0.25 g gum arabic; 5 ml 0.9% NaCl) (body weight in g x 15 = Volume of charcoal solution in μL) was administered intra gastric, by gavage, 10 min before killing, and migration of charcoal solution from the pylorus along the small intestine was measured. The small intestine was removed and gently, without tension, stretched. The migration of the charcoal was measured with a ruler. Intestinal and liver tissue was snap-frozen for RNA and protein extraction.

**Table 1:** Diet composition (% w/w)

	**w/w**	**C**	**C+ TRP**	**SFA**	**SFA +TRP**	**MUFA**	**MUFA + TRP**	**PUFA**
Casein	%	23.00	23.00	24.00	24.00	24.00	24.00	24.00
Corn starch, pre-gelatinized	%	34.90	34.90	30.00	30.00	30.00	30.00	30.00
Maltodextrin	%	14.28	14.04	8.28	8.04	8.28	8.04	8.28
Sucrose	%	5.20	5.20	10.00	10.00	10.00	10.00	10.00
Cellulose powder	%	3.90	3.90	5.00	5.00	5.00	5.00	5.00
L-Cystine	%	0.30	0.30	0.30	0.30	0.30	0.30	0.30
L-Tryptophan	%	——–	0.24	——–	0.24	——–	0.24	——–
Vitamin premix^1^	%	1.20	1.20	1.20	1.20	1.20	1.20	1.20
Mineral & trace element premix^2^	%	6.00	6.00	6.00	6.00	6.00	6.00	6.00
Choline chloride (50%)	%	0.20	0.20	0.20	0.20	0.20	0.20	0.20
Butylated hydroxytoluene	%	0.02	0.02	0.02	0.02	0.02	0.02	0.02
Soy oil	%	11.00	11.00	——–	——–	——–	——–	——–
Beef tallow	%	——–	——–	15.00	15.00	——–		——–
Olive oil	%	——–	——–	——–	——–	15.00	15.00	——–
Safflower oil	%	——–	——–	——–	——–	——–	——–	15.00

C, Control; SFA, saturated fatty acids; TRP, tryptophan; MUFA, monounsaturated fatty acids; PUFA, polyunsaturated fatty acids.

^1^Vitamin premix supplied per kg of diet: retinol, 4.5 mg; cholecalciferol, 0.0375 mg; all-rac (DL-) α-tocopherol, 150 mg; menadione (as MNB), 20 mg; L-ascorbic acid, 30 mg; thiamin, 16 mg; riboflavin, 16 mg; pyridoxine, 18 mg; cobalamin, 0.03 mg; niacin, 49 mg; D-pantothenic acid, 56 mg; folic acid, 19 mg; D-biotin, 0.31 mg; inositol, 80 mg.

^2^Mineral & trace element premix supplied per kg of diet: Fe, 162 mg as ferrous fumarate; Mn, 98 mg as manganese sulphate; Zn, 64 mg as zinc sulphate; Cu, 14 mg as copper sulphate; I, 1.2 mg as calcium iodate, Se, 0.11 mg as sodium selenite; and Co, 0.12 mg as cobalt carbonate.

**Table 2:** Fatty acid composition of the different experimental diets (% wt/wt)

**Fatty acids**	**wt/wt**	**Soy oil (C)**	**Beef tallow (SFA)**	**Olive oil (MUFA)**	**Safflower oil (PUFA)**
Energy content (kJ/g)	%	17.2	17.6	17.6	17.6
C 12:0	%	-	0.02	-	-
C 14:0	%	0.01	0.5	0.01	0.01
C 16:0	%	1.25	3.76	1.65	0.98
C 16:1	%	0.01	-	-	-
C 18:0	%	0.45	2.65	0.43	0.37
C 18:1	%	2.30	5.50	10.44	1.59
C 18:2	%	5.13	0.38	1.24	11.27
C 18:3	%	0.79	-	-	-
C 20:0	%	0.03	-	-	-
Others^1^	%	1.03	1.60	1.39	0.62
Σ SFA	%	1.74	7.14	1.75	1.45
Σ MUFA	%	2.31	5.88	10.62	1.66
Σ PUFA	%	5.92	0.38	1.24	11.27

*Abbreviations:* C, Control; *SFA*, saturated fatty acids; *MUFA*, monounsaturated fatty acids; *PUFA*, polyunsaturated fatty acids. ^1^Not specified fatty acids.

### Hepatic lipid analysis and histology

Liver tissue pieces (50–100 mg) were homogenized in ice-cold 2x PBS and lipids were extracted. Triglycerides were assessed with a kit (Randox, Krefeld, Germany). Values were normalized to protein concentration, determined by Bradford assay, in liver homogenates (Bio-Rad Laboratories, Munich, Germany).

To determine hepatic lipid accumulation, frozen sections of liver (10 μm) were stained with Oil Red O and counterstained with haematoxylin (Sigma, Steinheim, Germany). Representative photomicrographs were captured at a 400x magnification using Axio Vert 200 M, (Zeiss, Jena, Germany).

Liver histology was assessed in liver sections using haematoxylin and eosin staining.

### Endotoxin assay

Portal plasma samples were heated at 73°C for 20 min. Endotoxin levels were determined using a limulus amebocyte lysate assay kit (concentration range of 0.015–1.2 EU/mL)(Charles River, Wilmington, MA).

### Protein expression and gene expression

Protein was prepared and antibodies were applied as described earlier [17]. Semi-quantitative real-time PCR analyses were performed using SsoFast EvaGreen Supermix (BioRad Laboratories, Munich, Germany) and were carried out in an iCycler (BioRad Laboratories, Munich, Germany) with 40 cycles of a two-step PCR (denaturation 95°C for 35 s, denaturation 95°C for 5 s, annealing/extension 62°C for 10 s). MyD88, TNF-α and β-actin primer sequences were used as previously described [17]. Tryptophan hydroxylase 1 (THP1) primer sequence: forward CAT CAG CCG AGA ACA GTT GA; reverse TTC GGA TCC ATA CAA CAG CA.

### Measurement of the serotonin content in the duodenum

Frozen duodenum was homogenized in 0.05 M HCL with 0.1% ascorbic acid. 5-HT content (in ng/mg weight) was determined using an enzyme immunoassay kit (sensitivity 2.68 ng/mL) (IBL, Hamburg, Germany).

### Statistical analyses

All results are presented as means ± SEM. Effects of fat quantity in the diet were evaluated by comparing the results in the control group receiving an 11% fat diet with those in the fatty acid enriched groups receiving 15% fat diets. To do this, the means of the control group and the means of the combined fatty acid enriched groups were calculated and compared using the t-test, or t-test with the Welch’s correction, if variances differed significantly. One-way ANOVA analysis and Tukey’s post hoc test or Bartlett’s test for equal variances was used to compare the fat quality within the three groups receiving fatty acid enriched diets (SFA, MUFA, PUFA). One-way ANOVA was also used to analyze the effects of TRP in animals receiving either SFA or MUFA enriched diets. If the Bartlett’s test showed no equal variances, the Kruskal Wallis test with Dunn’s post hoc test was used. A *P* value < 0.05 was determined as the level of significance prior to study start. The software GraphPad Prism 5 (GraphPad Software, La Jolla, CA) was used for calculation and graph design.

## Results

### Fatty acids affect fat accumulation in the liver

**Figure 1:**
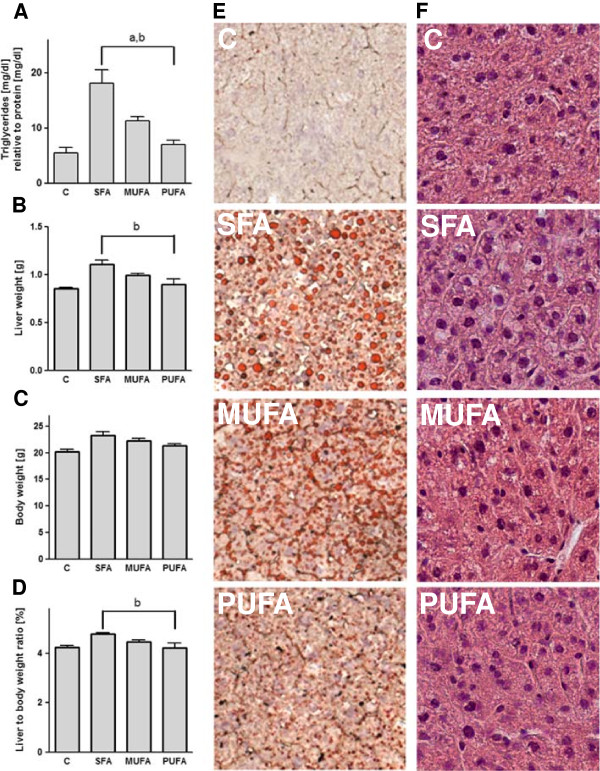
**Saturated fatty acids increase fatty acid accumulation in the liver.** Triglycerides **(A)** were measured with a colorimetric kit in the liver. Liver weight **(B)**, final body weight **(C)** and liver to body ratio **(D)** are shown. Oil-Red-O staining shows representative fatty acid accumulation in the liver **(E)**. Haematoxylin eosin staining shows liver histology **(F). **^a^*P* < 0.05 ANOVA result for the effect of the MUFA diet compared to SFA; ^b^PUFA compared to SFA. Means ± SEM, *n* = 5. *Abbreviations:* C, control; SFA, saturated fatty acids; MUFA, monounsaturated fatty acids; PUFA, polyunsaturated fatty acids.

Enhancing the fat content in the diet from 11% to 15% did not change significantly hepatic triglyceride levels. However, fat quality had a pronounced effect. SFA diet, compared to PUFA and to MUFA diet, caused higher triglyceride levels in the liver (*P* < 0.01, Figure 1A), and a higher liver weight (*P* < 0.05, Figure 1B). Although body weight was not influenced in mice fed the SFA, MUFA or PUFA diet (Figure 1C), we measured an increased liver to body weight ratio (*P* < 0.01, Figure 1D). We supported our results with representative staining of fat accumulation in the liver, showing an increase in hepatic fat feeding SFA or MUFA compared to PUFA enriched or control diet (Figure 1E). In addition, we observed a higher degree of steatosis and ballooning cells in SFA fed mice compared to a lower degree of steatosis and ballooning cells in MUFA and almost normal liver appearance in PUFA fed mice (Figure 1F). However, mice fed with the PUFA enriched diet had a similar elevated food intake as mice fed with the SFA enriched diet in contrast to MUFA fed mice (*P* < 0.05, Table 3). Overall water intake was reduced due to fat quantity. Mice fed the diets enriched with fatty acids drank less water compared to the control group (*P* < 0.001). Fat quality had an impact on water consumption, respectively. SFA and MUFA fed mice showed reduced water consumption (*P* < 0.01) compared to PUFA fed mice (Table 3).

**Table 3:** Effect of fatty acids on food and water intake as well as intestinal motility

**Diets**	**C**	**SFA**	**MUFA**	**PUFA**
*n*	5	5	5	5
Food intake (kcal/wk)	63.77 ± 0.43	80.52 ± 2.36	70.43 ± 1.63^a^	79.32 ± 1.34^b^
Water intake (ml/wk)	25.61 ± 0.19***	16.97 ± 0.40	16.92 ± 0.41	22.58 ± 0.29^a,b^
Intestinal motility (distance in cm)	8.72 ± 2.06	14.78 ± 1.57	11.70 ± 2.35	14.14 ± 2.20

Means ± SEM from *n* = 5 mice per group. ^a^*P* < 0.05 food intake compared to SFA, ^b^*P* < 0.05, food intake compared to MUFA. ^***^P < 0.001 water intake compared to mean of fatty acid enriched diet groups. ^a,b^*P* < 0.01 water intake compared to SFA and MUFA. *Abbreviations:* C, Control; SFA, saturated fatty acids; MUFA, monounsaturated fatty acids; PUFA, polyunsaturated fatty acids.

### Influence of fatty acids on portal endotoxin, hepatic MyD88 and intestinal tight junctions

**Figure 2:**
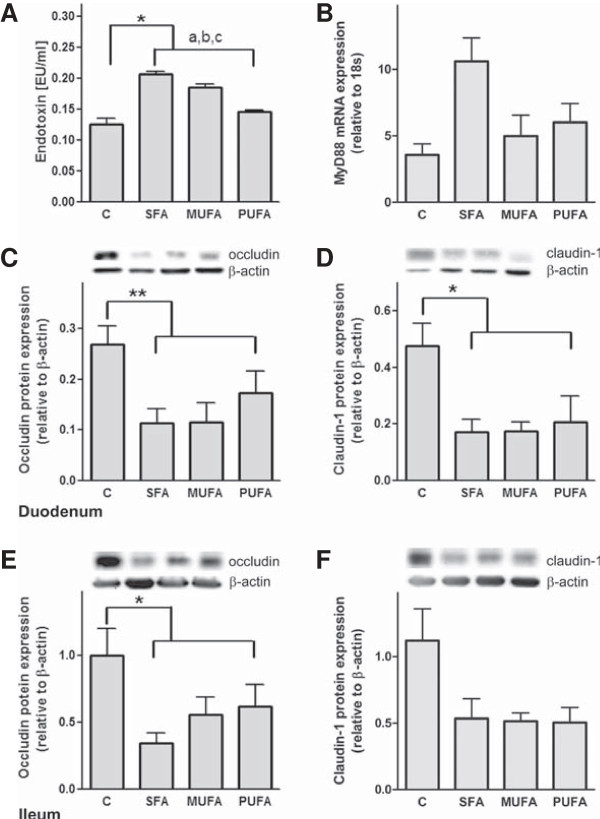
**Influence of fatty acids on portal endotoxin, hepatic MyD88 and intestinal tight junction proteins.** Portal endotoxin concentration **(A)** and MyD88 mRNA concentration **(B)** in the liver are shown. Representative western blots of occludin **(C/E)** and claudin-1 **(D/F)** in the small intestine with quantitative analysis of protein expression are shown. ^a^*P* < 0.05 ANOVA result for effect of different diets compared to C; ^b^ compared to SFA, ^c^compared to MUFA. Means ± SEM, *n* = 5. *Abbreviations:* C, control; SFA, saturated fatty acids; MUFA, monounsaturated fatty acids; PUFA, polyunsaturated fatty acids; MyD88, myeloid differentiation primary response gene 88.

We show that portal endotoxin levels were increased in mice fed the diets enriched with fatty acids compared to control mice (*P* < 0.05, Figure 2A). Fat quality, here SFA and MUFA diets, increased endotoxin concentration, respectively (*P* < 0.001, Figure 2A). MyD88 is known as a marker for endotoxin translocation and the subsequent stimulation of TNF-α [26]. In our study MyD88 was not influenced by fat quantity or quality neither on mRNA (Figure 2B) nor on protein level (data not shown).

In previous studies, we and others could show that a decreased expression of the tight junction protein occludin in the small intestine is associated with an increased translocation of endotoxin from the small intestine to the liver [27,28].

Here, we show a decreased occludin protein concentration in the duodenum (*P* < 0.01, Figure 2C) and the ileum (*P <* 0.05, Figure 2E) of mice fed the diets enriched with fatty acids. In addition, claudin-1 protein levels were reduced in the duodenum (*P <* 0.05, Figure 2D) but not in the ileum (Figure 2F) of mice fed the diets enriched with fatty acids.

### Effect of fatty acids on the serotonergic system

**Figure 3:**
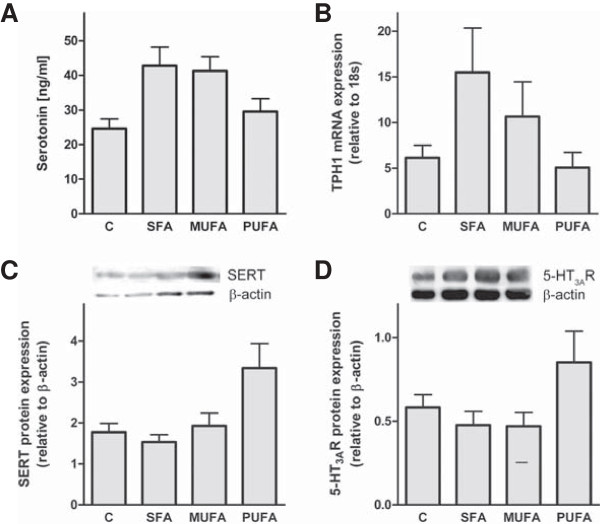
**Dietary saturated and monounsaturated fatty acids seem not to influence the intestinal serotonergic system.** Quantitative analysis of the 5-HT content and the TPH1 mRNA expression in the duodenum **(A/B)**. Representative western blots of SERT and 5-HT_3A_R in the duodenum and quantitative analysis of protein expression are shown **(C/D)**. ^a^*P* < 0.05 ANOVA result for effect of different diets compared to C; ^b^ compared to SFA. Means ± SEM, *n* = 5. *Abbreviations:* C, control; SFA, saturated fatty acids; MUFA, monounsaturated fatty acids; PUFA, polyunsaturated fatty acids; 5-HT, serotonin; 5-HT_3A_R, 5-HT receptor 3A; SERT, 5-HT reuptake transporter; TPH1, Tryptophanhydroxylase 1.

5-HT, which is produced at high amounts in the GI tract, is involved in the regulation of intestinal motility and possibly permeability [7,17,29]. Hence, we quantified 5-HT concentration, TPH1 mRNA expression, as well as 5-HT transporter (SERT), and 5-HT_3A_R protein content in the duodenum of mice (Figure 3). We found no significant change of intestinal 5-HT neither in fatty acid enriched diet fed mice in comparison to control mice nor between the diverse fatty acid groups (Figure 3A). Similarly, TPH1, the rate limiting enzyme during 5-HT synthesis, showed a noticeable, but not significant effect neither regarding the fatty acid enriched diets in comparison to control mice nor the fatty acid quality (Figure 3B). SERT protein expression was not affected in mice fed with fatty acid enriched diets compared to control diet or influenced by fatty acid quality, respectively (Figure 3C). However, SERT protein expression was nearly twice as high in mice fed a diet enriched with PUFA compared to mice fed a SFA enriched diet (Figure 3C). A similar but not significant tendency was shown for the 5-HT_3A_R protein expression (Figure 3D). Motility, although slightly elevated in mice fed the fatty acid enriched diets, was not significantly influenced by fat quantity or quality (Table 3).

### Influence of tryptophan and fatty acids on liver, intestine and the serotonergic system

**Figure 4:**
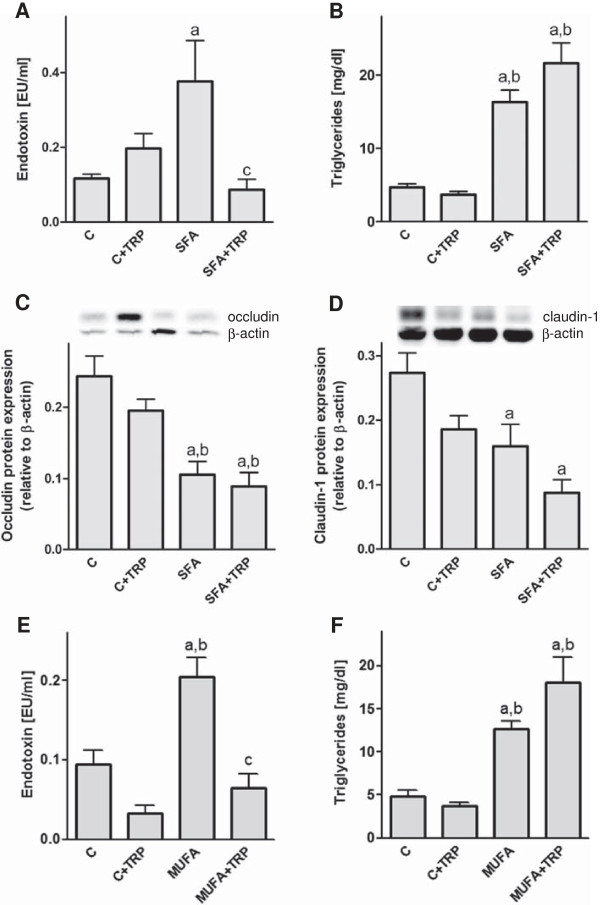
**Effect of TRP supplement and diets enriched with fatty acids on portal endotoxin, hepatic triglycerides and tight junction proteins.** Portal endotoxin **(A/E)**, triglycerides **(B/F)** and tight junction protein concentrations **(C/D)** were analyzed in mice fed TRP and SFA or MUFA diet. ^a^*P* < 0.05 ANOVA result for effect of the diets compared to C; ^b^*P* < 0.05 compared to C + TRP; ^c^*P* < 0.01 compared to SFA, ^c^*P* < 0.05 compared to MUFA. Means ± SEM, *n* = 5–6. *Abbreviations:* C, control; TRP, Tryptophan; SFA, saturated fatty acids, MUFA, monounsaturated fatty acids.

Interestingly, our second experiment feeding TRP as a supplement showed a reduction (*P* < 0.01) of the SFA induced increase (*P* < 0.05) in portal endotoxin concentration (Figure 4A,E). However, the elevated hepatic triglycerides and the significant loss of the tight junction proteins occludin and claudin-1, in the duodenum after feeding the SFA enriched diet, was not normalized by TRP (Figure 4B, C, D). Similarly, TRP supplement neither influenced food nor water intake in SFA enriched or control diet fed mice. In mice fed with the MUFA enriched diet TRP supplement had similar effects as shown for the SFA diet, reducing portal endotoxin (*P* < 0.05) but not hepatic triglyceride concentration (*P* < 0.05)(Figure 4E, F). Motility slightly increased in control mice after feeding TRP (Table 4).

**Table 4:** Effect of tryptophan in combination with fatty acids on food and water intake as well as motility

**Diets**	**C**	**C + TRP**	**SFA**	**SFA + TRP**
*n*	6	6	6	6
Food intake (kcal/wk)	80.18 ± 1.52	91.98 ± 2.10	98.58 ± 1.23^a^	117.88 ± 4.24^a,b^
Water intake (ml/wk)	26.42 ± 0.88	27.21 ± 2.44	18.68 ± 0.77^a^	21.02 ± 1.22^b^
Intestinal motility (distance in cm)	12.09 ± 3.37	18.93 ± 1.32^a^	16.41 ±1.63	17.12 ± 2.12

Means ± SEM from *n* = 6 mice per group. ^a^*P* < 0.05 compared to C, ^b^*P* < 0.05 compared to C + TRP. *Abbreviations*: C, control; SFA, saturated fatty acids; TRP, tryptophan.

## Discussion

Diets enriched with particular fatty acids such as SFA and MUFA, but not PUFA, cause the induction of fatty liver disease in C57BL/6 mice when compared to mice fed with a control diet. We selected this voluntary feeding model, instead of genetic models of obesity or forced feeding models because our model reflects best the human situation wherein primarily eating habits, besides genetic constellations cause NAFLD and obesity. Moreover, our approach allows the comparison of the effect of fatty acid quantity and quality on liver pathology, intestinal permeability and the serotonergic system.

Among the three diets, the SFA enriched diet had the most pronounced effects on fatty liver disease and weight gain, followed by the MUFA enriched diet. Earlier studies also reported that SFA and MUFA, but not PUFA induce changes in body weight and composition [30,31]. On the other hand MUFA are thought to ameliorate the plasma lipid profile in humans [32]. However, the underlying mechanisms still remain unclear [33].

The fact that PUFA ameliorate plasma triglycerides as well as low-density lipoprotein and high-density lipoprotein cholesterol in humans [34] underlines our findings that PUFA fed animals show only minor increase in triglycerides and hepatic fat accumulation in contrast to SFA and MUFA fed animals. One reason could be that PUFA expedite weight regulating or anti-inflammatory effects, for example in our study the slight up-regulation of the SERT reducing extra-cellular 5-HT levels. Furthermore, we cannot exclude that PUFA fed mice were still in their hyperphagic phase [35,36].

In contrast to our findings, another study showed an up-regulation of liver triglycerides by PUFA. Using a high-fat feeding model over 3 weeks in rats (n = 6; 59% fat-derived calories (PUFA = safflower), 23% carbohydrate-derived, 18% protein-derived) [37]. The difference might be due to the high amounts of PUFA that were fed in this study.

Most interestingly, the effects of fatty acid enriched diets on fatty liver disease and body weight may be partially related to the function of the gastrointestinal (GI) barrier. This is documented by our findings that fatty acid enriched diets reduced the protein expression of occludin in the small intestine what seems to result in enhanced endotoxin levels in the portal blood. Similarly, models of fatty liver disease, e.g. induced by dietary fructose or by lack of leptin showed that occludin protein expression is decreased and portal endotoxin levels are elevated in the small intestine [7,38]. For example Brun et al. (2007) reported that loss of the tight junction proteins occludin and zonula occludens 1 is associated with the development of NAFLD in *ob/ob* mice [11]. Taken together, there is increasing experimental evidence that the GI barrier plays a critical role in the pathogenesis of NAFLD [11,27,39]. We support this data showing elevated portal endotoxin concentration, what may be the trigger for the slightly but not significantly elevated MyD88 mRNA concentration in the liver of mice fed with the SFA enriched diet.

Furthermore, 5-HT seems to directly affect the expression of tight junction proteins in mammary gland cells [40]. We cannot support these *in vitro* findings since it is highly speculative to associate the reduced intestinal tight junction expression with the slightly but not significant increased intestinal 5-HT and TPH1 concentrations after feeding SFA and MUFA enriched diets. Another study reported a fibrogenic role for 5-HT in the liver by showing that 5-HT receptor antagonists attenuate the proliferative and enhance the apoptotic properties of hepatic stellate cells [41]. Indeed, we saw ballooning cells known as a pre-stage before the development of fibrosis in liver sections of SFA fed mice and to a lesser extent in the MUFA fed mice. However, if these findings are related to elevated hepatic serotonin levels needs to be proofed.

The tight junction protein expression was reduced in the duodenum and ileum after feeding the fatty acid enriched diets. Consequently, our data underlines a diet-induced GI barrier dysfunction and subsequent development of NAFLD.

Interestingly, our data suggest that endotoxin levels may be manipulated by TRP treatment. We clearly showed that SFA enriched diet with TRP supplement does ameliorate portal endotoxin concentration, but not, as expected, hepatic triglycerides or tight junction proteins. Here, TRP may lead to an increase of the enzyme indoleamine 2,3-dioxygenase in the intestinal epithelia that may cause growth arrest of several tryptophan-dependent microorganisms and is involved in repair processes within the epithelial barrier [42]. In contrast to the latter findings, it was shown that a high-fat and high-fructose diet supplemented with TRP aggravates hepatic steatosis in mice [43]. Nevertheless, it is not clear if this is a result of the possibly elevated 5-HT levels in the intestine [20,21] or due to a shift towards the pro-inflammatory tryptophan 2,3-dioxygenase/nicotinamide adenine dinucleotide pathway in the liver [25]. The underlying mechanisms for the here shown effects of TRP in combination with a high-fat diet remain unclear and need further analysis.

## Conclusion

In conclusion, our data clearly demonstrated that dietary fat components such as SFA and to a lesser extend MUFA are capable of modulating the intestinal barrier and liver fat accumulation but not necessarily the serotonergic system. Increased GI permeability may lead to an enhanced translocation of bacterial endotoxin to the liver and finally liver damage. Future prospects will be to elucidate the mechanisms by which SFA and MUFA, but not PUFA, cause the development of NAFLD.

## Competing interests

The authors declare that they have no competing interests.

## Authors’ contribution

SB, PE and SZ: designed the research. YR, MB, SH and AH: performed the research. YR, MB, SH and AH: analyzed the data. YR: wrote the paper. YR, SB, PE and SZ: revised the paper critically. All authors approved the final version of the manuscript.

## Pre-publication history

The pre-publication history for this paper can be accessed here:

http://www.biomedcentral.com/1471-230X/13/169/prepub
